# Early stage black pepper leaf disease prediction based on transfer learning using ConvNets

**DOI:** 10.1038/s41598-024-51884-0

**Published:** 2024-01-16

**Authors:** Anita S. Kini, K. V. Prema, Smitha N. Pai

**Affiliations:** 1 Department of Computer Science and Engineering, Manipal Institute of Technology, Manipal Academy of Higher Education (MAHE), Manipal, India; 2 Department of Computer Science and Engineering, Manipal Institute of Technology Bengaluru, Manipal Academy of Higher Education (MAHE), Manipal, India; 3 Department of Information and Communication Technology, Manipal Institute of Technology, Manipal Academy of Higher Education (MAHE), Manipal, India

**Keywords:** Electrical and electronic engineering, Computational models, Computational platforms and environments, Data acquisition, Data integration, Data processing, Image processing, Machine learning, Plant sciences, Environmental sciences, Energy science and technology, Engineering, Materials science, Mathematics and computing

## Abstract

Plants get exposed to diseases, insects and fungus. This causes heavy damages to crop resulting in various leaves diseases. Leaf diseases can be diagnosed at an early stage with the aid of a smart computer vision system and timely disease prevention can be targeted. Black pepper is a medicinal plant that is extensively used in Ayurvedic medicine because of its therapeutic properties. The proposed work represents an intelligent transfer learning technique through state-of-the-art deep learning implementation using convolutional neural network to predict the presence of prominent diseases in black pepper leaves. The ImageNet dataset available online is used for training deep neural network. Later, this trained network is utilized for the prediction of the newly developed black pepper leaf image dataset. The developed data set consist of real time leaf images, which are candidly taken from the fields and annotated under supervision of an expert. The leaf diseases considered are anthracnose, slow wilt, early stage phytophthora, phytophthora and yellowing. The hyperparameters chosen for tuning in to deep learning models are initial learning rates, optimization algorithm, image batches, epochs, validation and training data, etc. The accuracy obtained with 0.001 learning rate ranges from 99.1 to 99.7% for the Inception V3, GoogleNet, SqueezeNet and Resnet18 models. Proposed Resnet18 model outperforms all model with 99.67% accuracy. The resulting validation accuracy obtained using these models is high and the validation loss is low. This work represents improvement in agriculture and a cutting edge deep neural network method for early stage leaf disease identification and prediction. This is an approach using a deep learning network to predict early stage black pepper leaf diseases.

## Introduction

Worldwide crop losses estimated to be in the billions of dollars are attributed to plant diseases. Numerous agricultural diseases hamper the expansion of agriculture. Farmers anticipate diseases through visual symptoms in plants. In the most cases it is misjudged, misinterpreted, and frequently wrong. This leads to the unauthorised use of plant pesticides and chemicals. The catastrophic effect of plant disease is yielding loss and no harvest in some dreadful cases. Black pepper is a medicinal plant. Black pepper is referred to as "black gold" in Ayurveda. It is the most crucial component in Indian and other cuisines and has numerous medicinal and health benefits. About 80% of people in developing countries, as well as about 3% of people in developed countries, are inclined towards using medicinal plants such as black pepper to treat their alignments^1^. This is the main ingredient in many ancient, unani, and ayurvedic medicines, which can cure many chronic diseases like diabetes, high blood pressure, the common cold, cough, etc. Black pepper plants are affected by diseases such as slow wilt, quick wilt, phytophthora, foot rot and yellowing etc. It is a native spice of south Karnataka, Tamil Nadu, Kerala, and some forest states of northeast India. The countries with the highest black pepper production, including India, are Vietnam and Indonesia. There is a growing demand and requirement for better technologies to automate disease detection. Currently, pathologists and agriculturalists rely on the traditional laboratory method and detect diseases with the naked eye. Computerized detection of plant diseases is crucial to identify and detect the primary signs of diseases so that they can be treated.

The scientific community has studied plant diseases extensively, mostly concentrating on the biological properties of plant illness. Plant diseases are unavoidable and have become a big challenge^2^. Crop production scales down due to influences of pests, fungal attacks, environmental effects such as an unexpected variation in the meteorological conditions, which escalate further losses. This encourages the practise of some cutting-edge techniques so that the diseases can be premeditated in the specified area for various disease variance. As a result, there will be less labour work and less time spent on conventional methods or waiting for an expert^3^. Plant diseases are dependent on specific environmental factors and soil culture. Intelligent techniques for identifying remote areas as well as adjacent areas would furnish a well-organised yield monitoring system for disease-ridden areas. For such circumstances, hyper-spectral sensors or thermography sensors could be brought into practise to limit human intervention and unreachable far-field areas^4^. The disease is visible because the crop has a patchy distribution throughout the field. For instance, studies of tomato leaves^5^, rice leaves^6^, and litchi leaves^7^ demonstrate how vulnerable plants are to infections^8^. The latest deep learning algorithm enhances the computational approach with precise disease identification and realistic output images.

Several real time computer vision systems have been recently developed using machine learning algorithms and other computational methods. Deep learning techniques are the fastest adopted techniques that help in analysis and feature extraction of dissimilar types of data^9^. Deep learning is a very popular methodology in almost all fields. Recently, it has gained tremendous popularity in agricultural domain because of its accurate results. Most of the researchers are training their data with an artificial neural network to get well-classified results^10^. Convolutional neural network (CNN) or ConvNet and artificial neural network (ANN) are the most successful deep learning techniques that has been associated with various image segmentation, feature extraction, and pattern identification or design recognition tasks.

**Figure 1:**
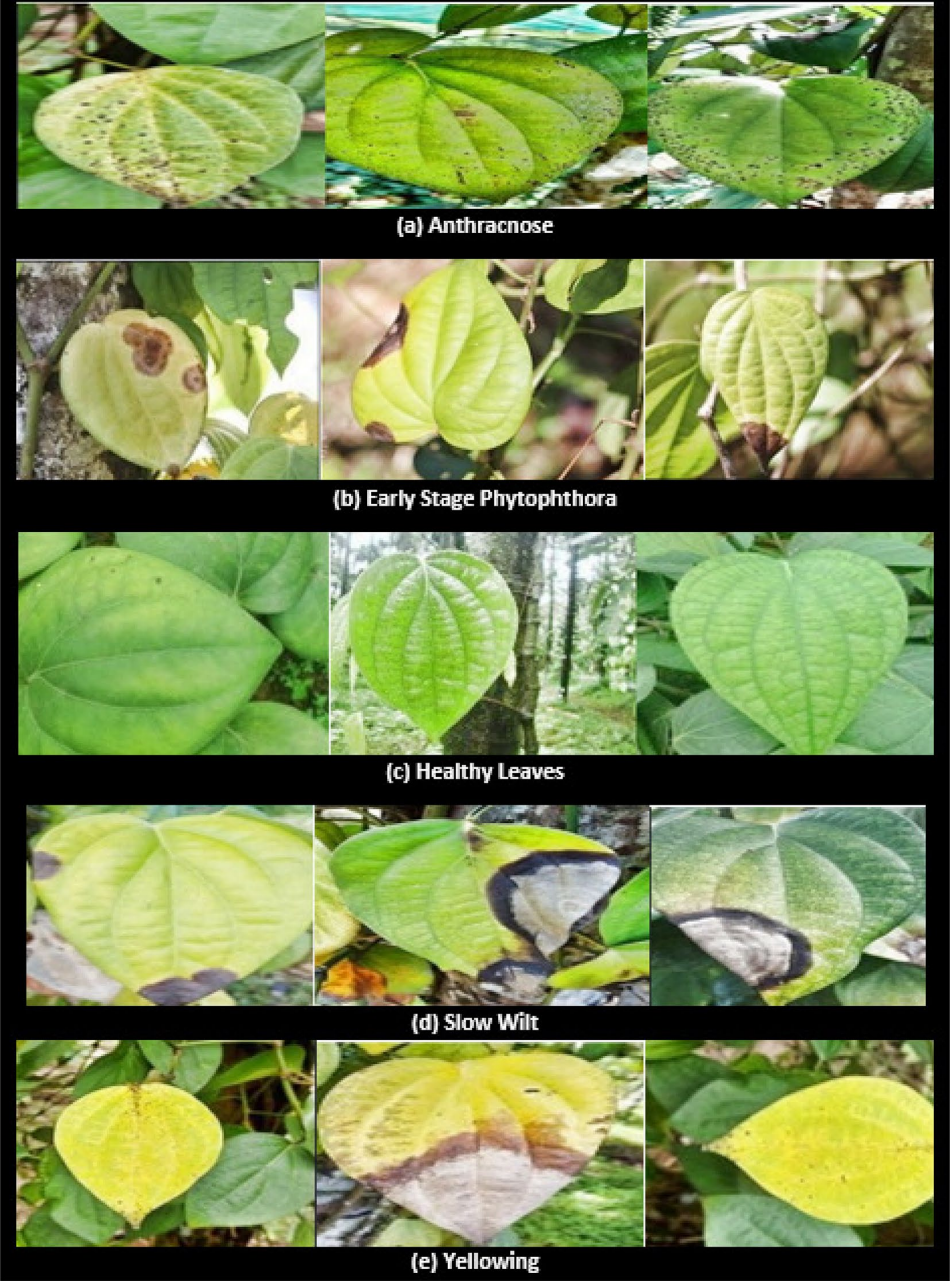
Black pepper leaf disease dataset.

The proposed work demonstrate implementation of stat-of -the-art deep learning in categorization of black pepper leaf diseases. The various diseases considered for the proposed work are depicted in Fig. 1. The dataset includes six prominent categories of black pepper leaf diseases those are Anthracnose, Early stage phytophthora, Slow wilt, Yellowing, Phytophthora and healthy leaves^11^. There have been numerous studies on different leaf and plant disease detection techniques. To our information, this is the original attempt to use transfer learning for the prediction of black pepper leaf diseases in early stage. The image dataset used in the experiment is collected from black pepper plantations from the districts of Madikeri, Karavali, Manipal, and Udupi region in southern Karnataka, India, and used as a “benchmark dataset” for the subsequent study.

The important contributions made by this work are as follows:

- The proposed approach uses real-time images of black pepper leaves captured by various high-end electronic equipment: a Nikon Coolpix, a Sony DSLR, and an Android phone.
- The benchmark dataset is created and annotated under the supervision of an agriculture scientist.
- The state-of-art deep neural network convolutional neural network algorithms are used along with transfer learning.
- A sophisticated high-end model for an early-stage black paper leaf disease is suggested.

The ensemble implementation of the state of art deep learning algorithm give better predictions and are better than working on any single deep neural network model. This is been observed with implementing model Inception V3 and implementing a combinative application of SqueezeNet, GoogleNet and ResNet-18 using transfer learning. The goal of any machine learning algorithm is to select the one model that can most precisely predict the intended outcome. Unlike single model strategies where we hope this is the best/most accurate prediction, we can construct ensemble approaches incorporate into account a wide range of models and average those models to build one final model. The ConvNets used are the faster image classification networks.

The article is arranged as follows: The motivation of the proposed work is mentioned in Section "Motivation", and related studies are discussed in Section "Related work". Section "Materials and methods" deals with materials and methods. In this section, various stages related to data acquisition, data preprocessing, image segmentation, convolutional networks, and transfer learning are discussed. Followed by Section "Experimental setup", where the experimental methodology is discussed with the help of a block diagram. Section "Result and discussions" includes results and discussion, and Sect. 7 includes conclusion and discussions followed by section "Future work", where current limitations and future work are discussed.

## Motivation

At present, there is a huge surge in the automation of everything existing in the world. There have been numerous studies on different leaf and plant disease detection techniques. Algorithms for machine learning are being applied to categorize leaf diseases. But the classification result is not accurate for a small dataset. There is a dearth of standard sets of evaluation criteria for leaf disease identification and classification. To the best of our knowledge, this is the first time that transfer learning has been used to detect black pepper leaf disease. This work proposes deep neural network learning implementation with high accuracy in early leaf disease predictions.

This experiment will encourage implementation of advanced technique in agriculture processes and reduce manual labor and overall cost of disease estimations. The data set created will also help novice farmer to learn about disease it’s variety and they will be able to apply preventive measure to save the crop loss at early stage itself.

Significance of the given research work:

1. Early stage leaf disease detection in black pepper.
2. Improved feature extraction technique for the black pepper leaf disease.
3. The given research work motivates contribution towards precision agriculture.
4. Encourage research in agriculture domain and usage of machine learning and deep learning for betterment of people.

## Related work

Recent progresses in deep neural networks have made it possible for researchers to significantly correct the precision of object identification and recognition techniques. The CNN models used in automatic image recognition systems are particularly useful for detecting the onset of diseases at different stages of plant development.

Barbedo^1^ discussed techniques for enhancing data that lessen the impact of the absence of image databases that can accurately depict the numerous illnesses and symptoms in a given image. But at the same time, they’re unable to replicate the majority of the practical diversity. This paper deals with specific lesions and patches instead of taking the leaf as a whole into account for this purpose. Too et al.^2^ elaborated that traditional machine learning approaches like support vector machine, multilayer perceptrons, decision trees and neural networks have historically been used to solve the challenges of autonomous disease recognition in plants. The study looks into how the quantity and diversity of datasets affect the efficacy of deep learning methods used in plant pathology. Barbedo^3^ elaborated that today there is a shift of technologies from traditional to convolutional neural networks, which are now the predominant approach that applies deep learning concepts.

Ganatra and Patel^5^ projected a prediction model for classifying and detecting plant leaf disease detection using machine learning and computer vision approaches. Preprocessing, segmentation, and extraction of attributes such as shape, color, texture, vein etc. are done on the raw image of a leaf. Thangaraj^8^ discussed one of the venerated deep learning techniques for accurately detecting plant disease with little to no plant image data, namely transfer learning. They used a deep learning neural network model built on transfer learning to recognize tomato leaf disease. The author has used available and real-time images of tomato plants to detect disease. Additionally, stochastic gradient descent (SGD), RMS prop optimizers, and adaptive moment estimation (Adam) are used to evaluate the performance of the suggested model. The result of the experiment demonstrated that the suggested model, which use transfer learning strategy, is efficient at automatically classifying tomato leaf diseases.

Ghosal et al.^9^ have worked on rice leaf, which is afflicted by a number of illnesses at different phases of its development. The authors created their own dataset of rice leaves due to a lack of available picture datasets and castoff transfer learning to create a model of deep learning. The suggested CNN architecture, which is based on VGG-16, was trained and tested using data gathered from the internet in addition to data gathered from the rice field. A total of 1509 rice leaf images were tested on 647 other images. The suggested architecture was a success. Cross-validation is advised to validate results in future work. Devaraj et al.^13^ frazzled on the importance of image processing in the arenas of agriculture and disease classification. Automatic disease detection is modelled using image segmentation and feature extraction. Upadhyay, et al.^14^ operated on brown spot diseases in paddy plants. The classification model based on CNN is trained and validated on the three major classes. Such as healthy leaves, developed spot leaves, and early stage leaves.

Saleem et al.^18^ have performed an analysis of deep learning and convolutional neural network optimizers. The work represents an appraisal of a hybrid model for plant disease detection. A total of 26 different diseases fall under 14 different plant species that were classified. The authors propose that the Xception architecture, when trained with the Adam optimizer, gives high accuracy. Fuentes et al.^27^ accentuated the importance of deep learning based on recent advancements towards object detection and recognition system accuracy. Cameras with different resolutions were used to gather real-time images of diseased tomato leaves. The authors used Single Shot Multibox (SSD), a region-based fully convolutional network, and a faster region-based convolutional neural network to complete their work. Single-shot multiboxes developed by the combination of two different region-based CNNs are discussed.

Numerous image processing applications have embraced the Bacterial Foraging Optimization (BFO) technique. This covers a wide range of tasks, such as multilevel color image segmentation, face and lip recognition, illness classification and identification from plant leaf images, pedestrian segmentation, and many more. Chouhan et al.^34^ have worked towards bacterial foraging optimization for plant disease detection. The significance of optimization techniques for image segmentation is demonstrated in this paper. Author determined that among other optimization techniques Bacterial Foraging Optimization is best, which is a member of the class of algorithms inspired by nature. When handling complex data, the hybridization of the bio-inspired algorithm also solves the issue with conventional methods. While dealing image processing.

Rakshit Khajuria et al.^35^ suggests that automatic detection methods are accurate, efficient, and save time when it comes to identifying plant diseases. In recent years, automation of plant disease detection has drawn more attention. in agriculture. Thus, the current approaches for leaf disease detection, segmentation, and identification have a great deal of room for improvement given the advancements in ANN, its family, and machine learning techniques. The benefits of using these ANNs in different ways to provide optimal or nearly optimal solutions are the main topic of this study. AI performs well in testing and training scenarios. ANN has a strong learning nature. These networks may route around missing data when they are scaled over several servers and computers. AI allows us to capture a larger image data set. Author didn’t review any data set in the literature. Author proposes that to identify, categorize, and detect leaf diseases, the interested writers might construct their own data collection.

Chouhan et al.^36^ conferred on data repository. This article has contributed to the understanding of plant development and management. For the stated reason, twelve environmentally and economically advantageous plants have been chosen. Images of these plants' leaves in both good and bad environments have been collected and alienating into two distinct classes. About 4503 photos of healthy as well as diseased are collected. In addition, writer anticipate that this work will help scientists and scholars create strategies for plant identification, categorization, growth monitoring, leaf disease diagnosis, and other tasks. The ultimate expectation is to have a better understanding of the plants that need to be planted and how to take care of them. Authors gathered the leaves of twelve distinct plants that are significant for the environment and economy. The goal of this endeavor is to gain a deeper comprehension of the plants that need to be grown and how best to maintain them. Writer anticipate that this research will significantly advance the development of automated techniques for comprehending, observing, and controlling plant growth. Additionally, they think that knowing enough about the plant that will be cultivated will play a big part in nurturing nature and need of the hour. Author also reasoned that plants possess an incredible resilience quality that allows them to heal themselves, which aids the ecosystem in combating climate change and carbon emissions.

Chouhan et al.^37^ presented an autonomous neural network (NN) based web aided leaf disease segmentation system for mango trees. There were four main components to the suggested system. A digital camera with web access is used to take real-time photos of the mango leaves, a scale-invariant feature transform technique to preprocess the images and extract features. Third, the most different features are used to optimize the bacterial foraging optimization technique for the NN's training. Lastly, the diseased region is extracted from the pictures of the mango leaves using the radial basis function of NN. An inexpensive, real-time web-enabled system that requires little human intervention for disease segmentation from leaf images is recommended. Researchers have introduced plant pathology's underlying technology, artificial intelligence, and smart devices that provide the foundation for precision or smart agriculture. Allowing neural networks and algorithms with natural inspiration to combine their learning and optimization powers for the purpose of identifying and categorizing diseases. Using the web-facilitated environment in addition to agricultural applications, such as managing weeds, estimating soil moisture, classifying plants using photos of their leaves, estimate crop growth, and manage fruit and flowers. Introducing the tools and technologies used in smart or precision agriculture, such as sensors, drones, smart cameras, and the internet of things. This article proposes an automated approach for separating the fungal disease known as anthracnose from the mango plant's leaves. The use of a camera equipped with Wi-Fi is advocated for the acquisition of leaf images in a real-time setting.

Chouhan et al.^38^ articulated that plant growth can be improved by the real-time decision support system, raising the crop's yield, quality, and economic worth. By monitoring plant development to maintain environmental balance, this also assists us in supporting the natural world. It has been demonstrated that computer vision techniques are crucial in many fields, including corporate analysis, agriculture, medicine, and the military. Precision or smart agriculture has demonstrated the dynamic nature of using digital image processing techniques to simulate human vision. To improve crop yield and quality, the concept discussed has made an approach possible to automatically prevent and monitor plants, cultivate, manage diseases, control water, and more. In this research, author have conducted a survey of the number of articles that use soft computing and computer vision techniques to identify and classify plant illnesses from their leaves. This work showcases the most recent ideas, theories, and applications related to soft computing and digital image processing techniques. Each of the several results has been covered separately in the given paper. Different findings, grouping them according to the stages involved in digital image processing have been taken care. The main issue discussed in the survey is the standard database's accessibility about specific plant and disease conditions. The inaccessibility of the database discourages scholars and researchers from working in this area. The issue of database acquisition is another hurdle. The process of collecting real-time databases from the field in a variety of atmospheric circumstances is exceedingly challenging and time-consuming. The preparation of the image is another problem. Real-time images have several invariabilities, such as varied textures, background features, and angles at which the image is captured, among other things. Other issues include choosing the actual field area for taking the picture, choosing the plant, choosing the portion of the plant that is diseased, and choosing the disease itself. Another issue that needs to be addressed by the author is the choice of diseases and their symptoms that distinguish one from the other. The selection of a technique and ideas for creating a suitable system that performs better, takes less time, and is more affordable comes next. There are several relative issues dealing with real time raw images. The difficulty associated with uncertain real time data and implementation of smart computer vision techniques is applauded.

## Materials and methods

### Image acquisition

**Figure 2:**
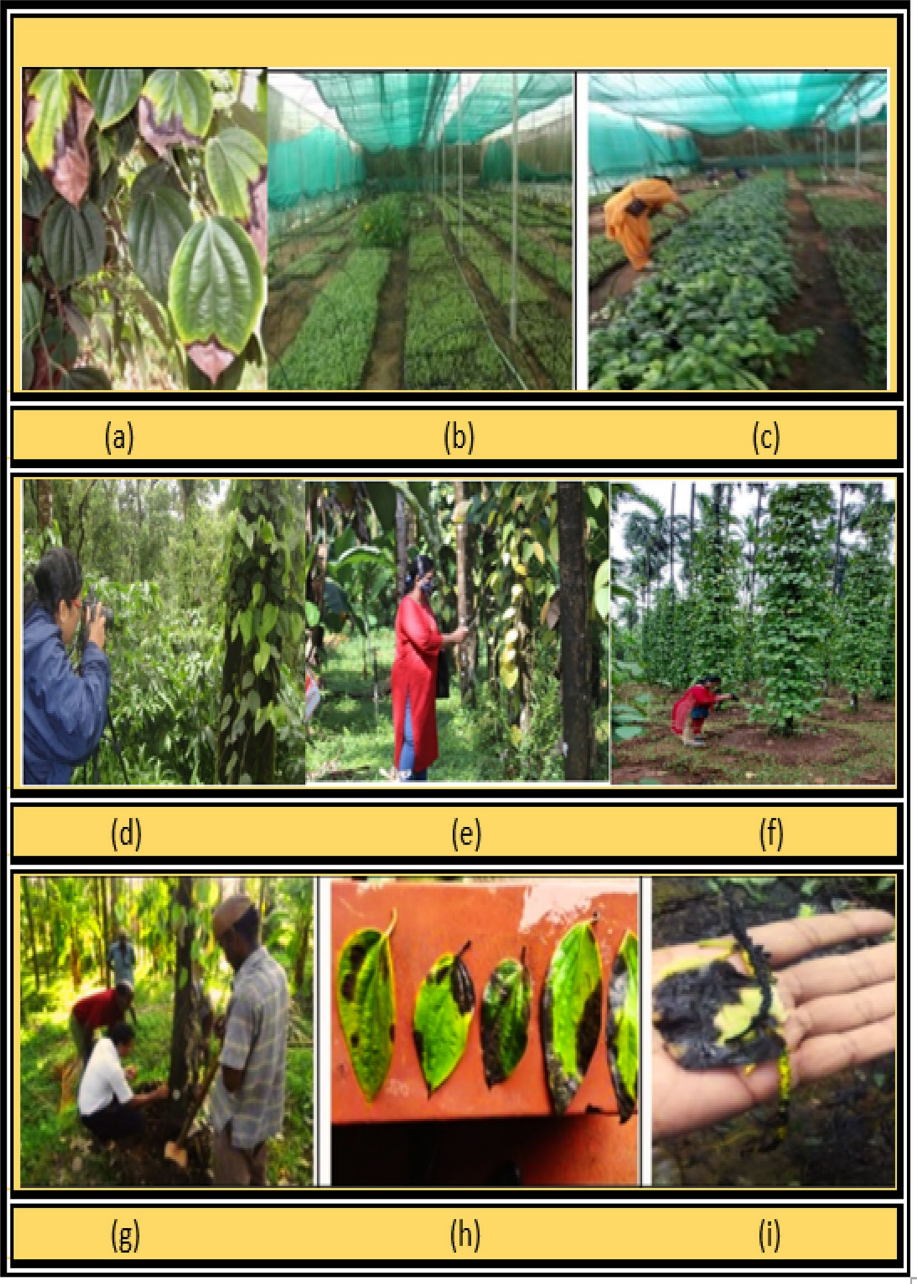
(**a**–**i**) Field scouting in the black pepper field with an expert.

In the proposed research work, an expert-annotated benchmark dataset is considered. Data were directly captured from the field with the aid of high-end electronic devices: a Nikon Coolpix, a Sony DSLR, and an Android phone. Images captured by high-resolution cameras in a variety of dimensions are transformed to a uniform size, typically 300 × 300 pixels. Since the gathered images have different sizes, it is vital to make them all the same size. A collection of 1800 distinct photos of diseased and healthy black pepper leaves were considered for testing. These pictures were taken at random for both healthy and unhealthy leaves. For each disease type, almost 300 images were collected. Data collected from various region in Karnataka, India is depicted in Fig. 2. Figure 2a–i shows field scouting in the black pepper field with an expert.

### Image preprocessing

The visual spectrum is sensitive to the cameras used for regular photographs. There are problems with recording live photographs, including the absence of a distinct and detailed border, an ambiguous background, symptoms of various disorders, variations in the lighting conditions, an unexpected brightness, a dull or cluttered background, and others. These elements collectively have a significant impact on image analysis. Real photos need preprocessing in order to fully analyze them.

**Figure 3:**
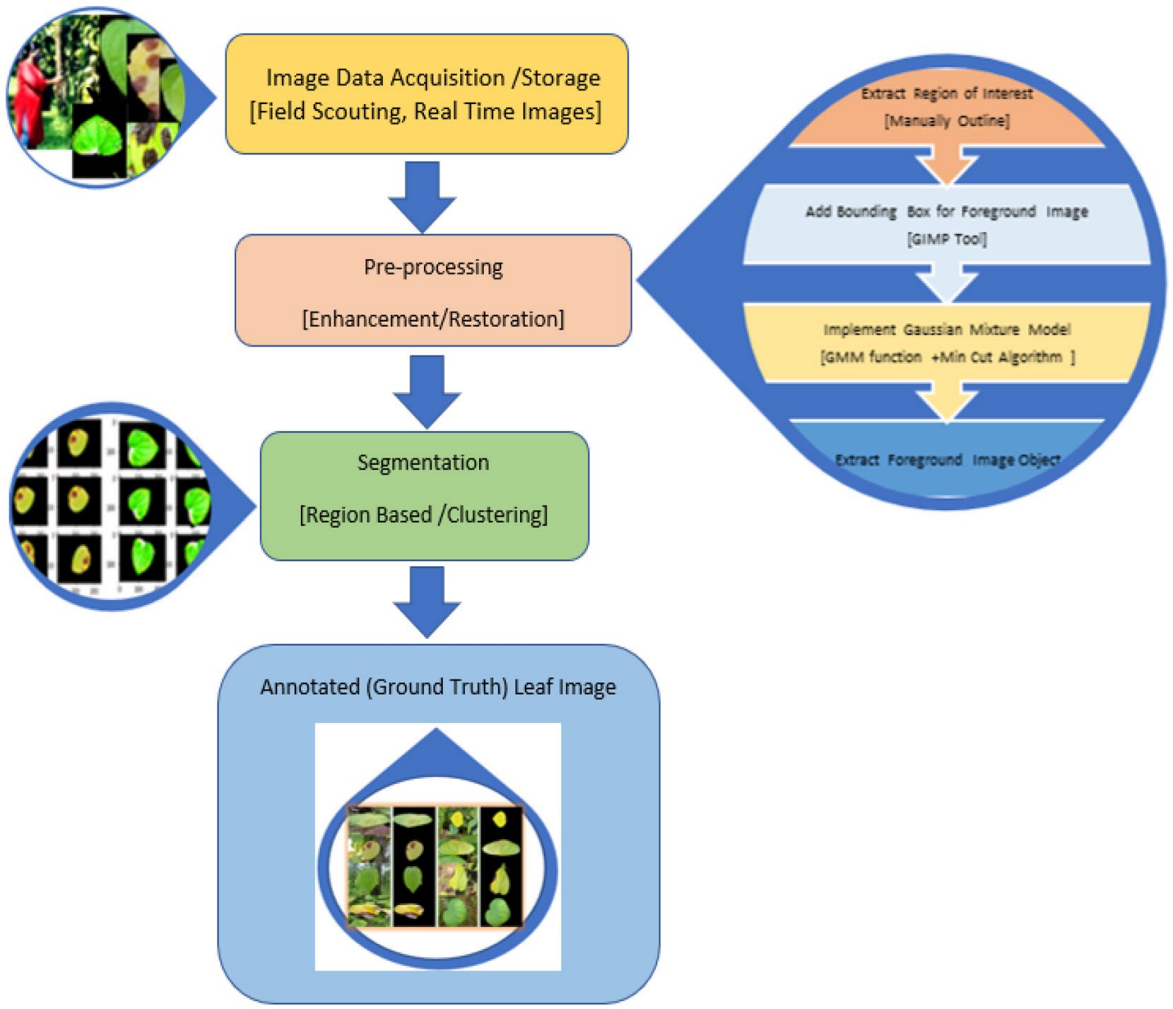
Data acquisition and annotation.

**Figure 4:**
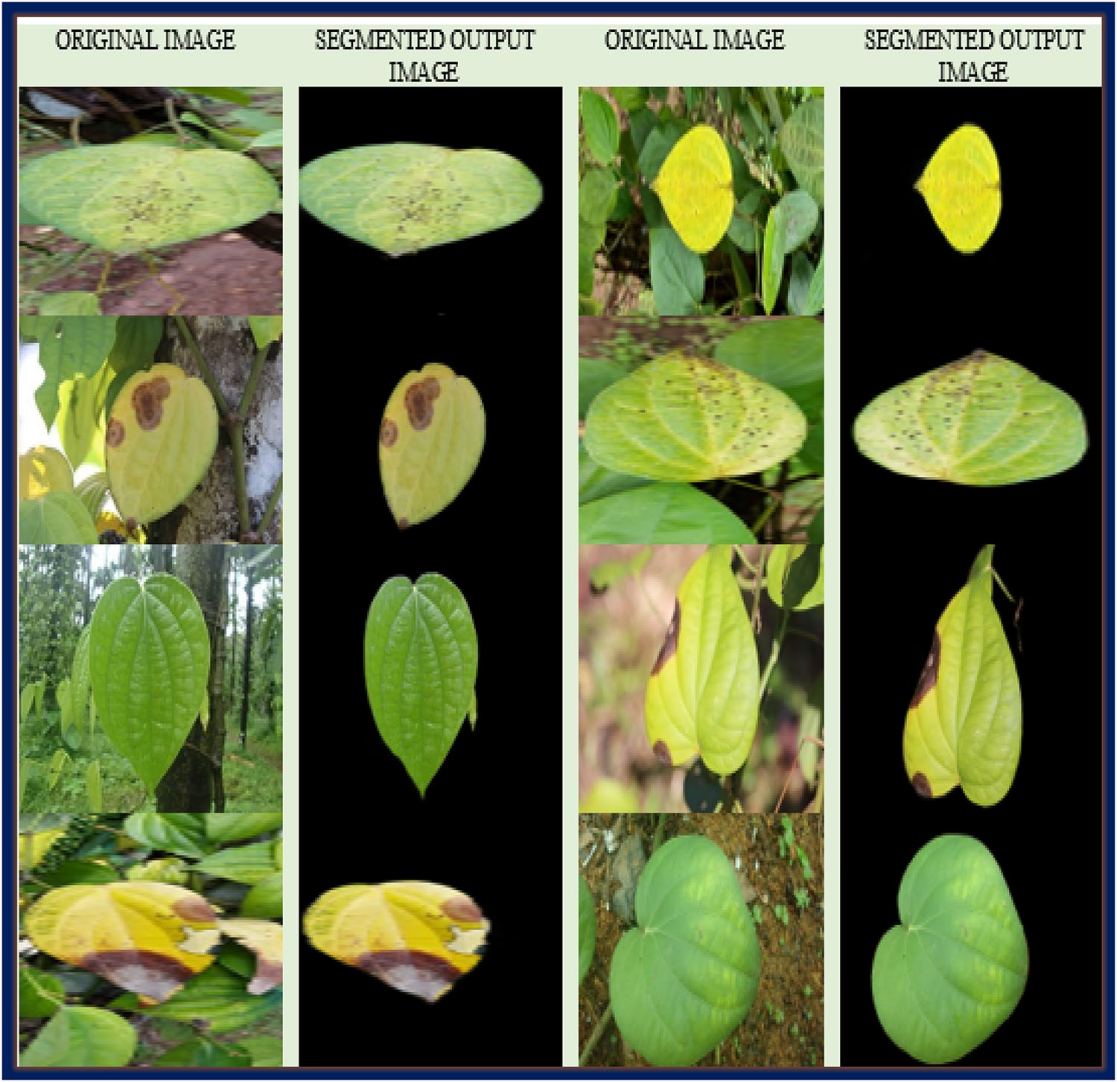
Sample annotated (ground truth) Images.

A ground truth image that is the region of interest is obtained using the grab cut algorithm. The initial step to performing the above-stated task was to mark the region of interest. To achieve this, we have used online image processing, the GIMP tool. Where we use a bounding box to mark the image. Everything outside ROI (bounding box) is considered as background and masked as zero, which is black. Next, these images are used MATLAB R2023a for further preprocessing. The code is written in python, importing suitable libraries. The Gaussian mixture model (GMM) is used to filter the background and foreground. GMM learns the pixel to separate background and foreground, considering the color pixels and the area outside the rectangular block to label the image to provide the ground truth image. Later, the division is made by implementing grab-cut algorithm, where the clustering method is used by clustering all the forefront images to one node known as the source node and all the background pixels to another node known as the sink node^11^. These pixels are hard-labelled based on their closeness to the source node as foreground and their sink node as background. The weight among the pixels is defined by the edges defined by the rectangle block. This decides the probability of grouping pixels in each terminal. The min-cut (minimum-cut) technique is used to further partition the image graph into distinct portions using the minimum cost function. Considering the probability of the pixels being next to the source node or sink node, the image is segmented, and finally, labelled annotated image data is obtained. Figure 3 depicts complete pipeline showing image processing-processing and data annotation. Figure 4. show sample Annotated (ground truth) images.

### Image augmentation

**Figure 5:**
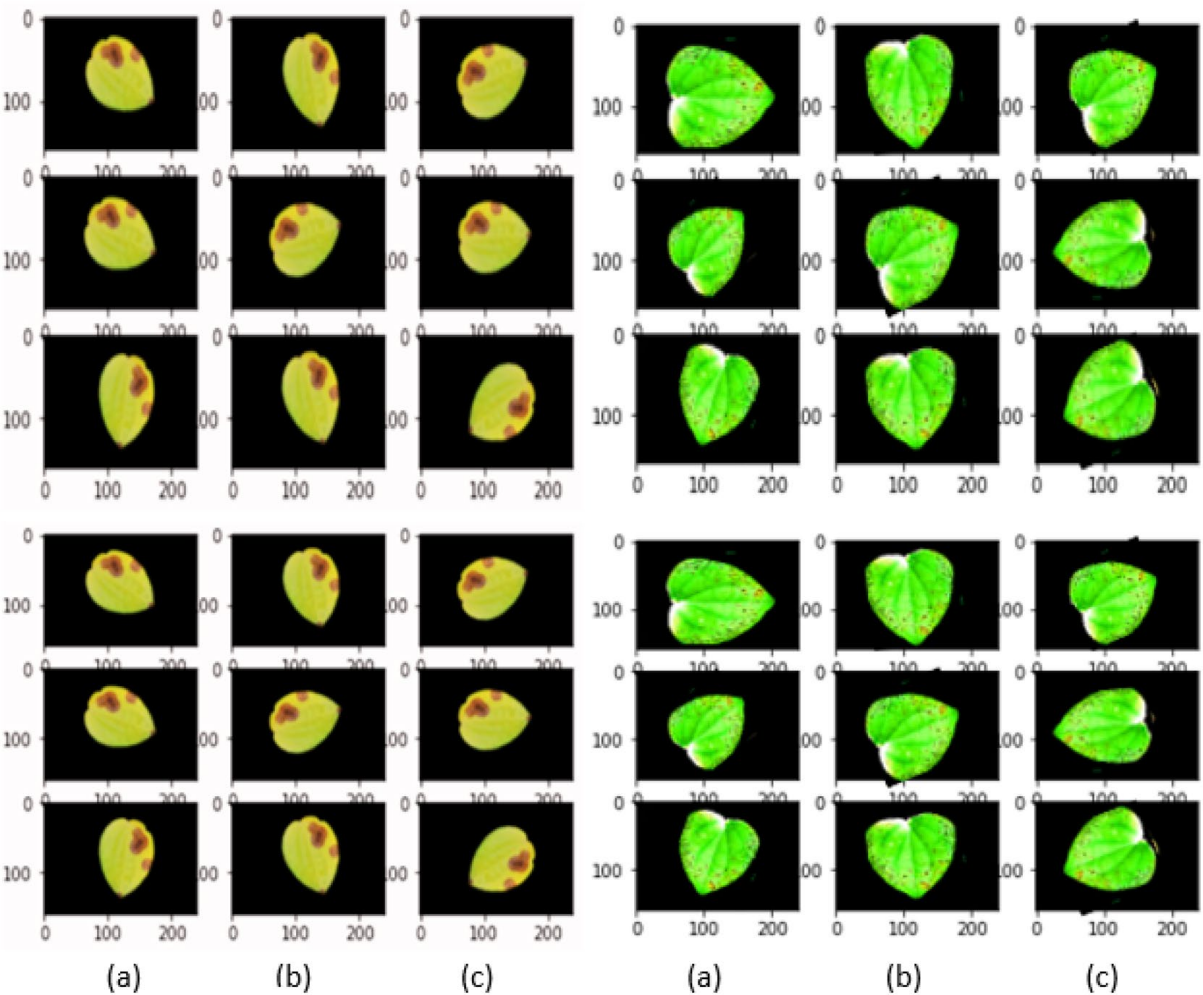
Image augmentation (**a**) Random reflection x axis (**b**) Random rotation (**c**) Random rescaling.

Image augmentation is a method where the images are modified into a new form of the similar image. This is done to expand the training data set. Augmentation is used to represent a given image in various versions. Such that the images are flipped, rotated, and tilted to add extra records to the given database^12^. To artificially increase the training dataset, the given data is vertically rotated on the x-axis, modified at a 90-degree angle, and rescaled as elucidated in some sample in Fig. 5. This is achieved using python’s built-in functions while training a deep neural network.

### Convolutional neural networks

**Figure 6:**
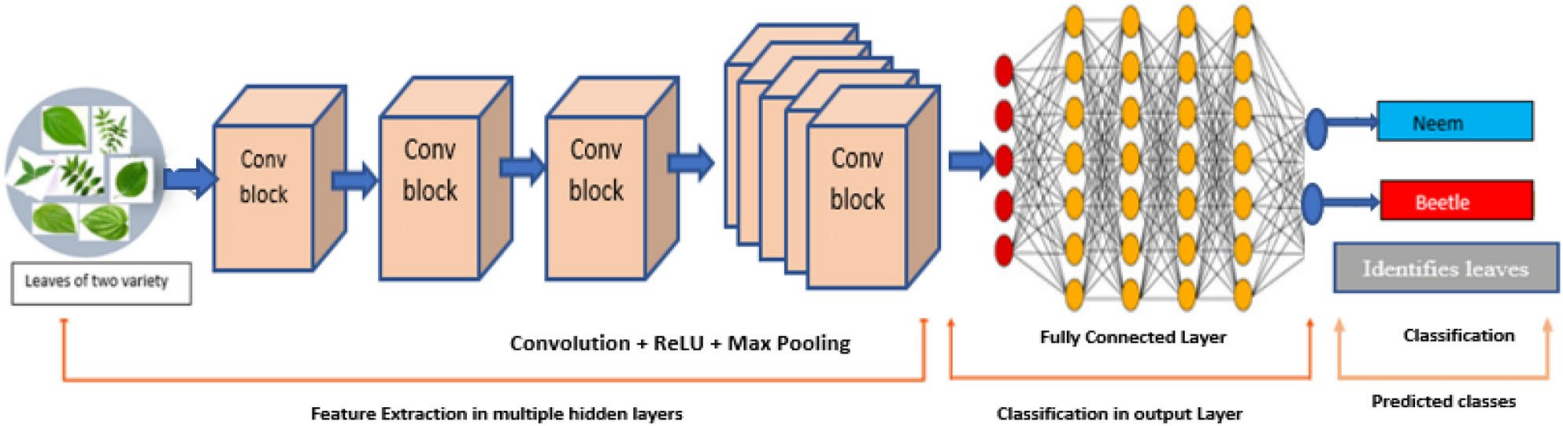
CNN architecture.

Feature extraction is the main reason for using machine learning for data organization. Convolutional Neural Network (CNN) is a type of machine learning algorithm that has been widely used for image categorization in recent years. These collection of algorithms in neural networks are termed as deep learning algorithms. These algorithms are the extensions of neural network processes. The convolutional neural network is utilized to automatically train the diseases^13^. Such systems are real-time deployable. Convolutional layers in CNN are followed by a pooling layer, an intermediate layer and further layers. There is an activation function in the pooling layer and between each set of the convolutional layer, Fig. 6. The input image was processed through a filter by the convolutional layer primarily, which modifies the pixel intensities^14^. The activation function helps in determining neurons’ states, which also sends a signal to the subsequent linked neuron in the higher layers. The convolution responses are shrunk down to a smaller dimension using the pooling layer. This layer can be used with a variety of pooling techniques, including maximum, minimum, average, and other types^15,16^. We have implemented a CNN to classify the black pepper plant leaf images based on disease categories and implemented max pooling (maximum) for multi class classification.

#### Inception V3

The recommended Inception V3 neural network architecture, is 22 layers deep with 27 pooling layers encompassed, depicting the stepwise execution details. It consists of a total of nine linearly stacked inception modules. Terminals of the inception module are connected to the global average pooling layer. The deep neural network is designed at the succeeding layer by replacing the last few layers, including the final layer^17^. For these layers, the learning rate is set high, to incorporate a faster learning rate into the newly formed (higher-level) layers. Inception V3 is employed to classify diseases in black pepper leaves effectively.

#### Google Net

Google net was designed and proposed by google in collaboration with numerous universities. Google net consists of many different kind of 1 × 1 convolution, average pooling to create deeper architecture. The convolutional network is used to reduce the number of parameters related to weight and bias so that small network can be build up^18^. The global average pooling used at the end” of this network, averages a feature map of 7 × 7 to 1 × 1. Thereby decreasing trainable parameters to 0 while improving accuracy of high class. The accuracy of google net is much more improved than its predecessors.

#### SqueezeNet

The squeezenet architecture is also designed to reduce the parameter of the original data matrix. It reduces the dimensions by using 1 × 1 convolution by the using the design strategy that normally squeezes the parameter and the design is termed as fire modules. Squeeze Net architecture are based on embedded system which are highly resource inhibited operations. The main filter used in this system is of size 1 × 1 and not 3 × 3 that is used in most of the deep learning models. The fire model consists of squeeze convolutional layer which has filter of size 1 × 1 this is further expanded and feed into other filters that are expandable s this would expand to mixture of 1 × 1and 3 × 3 filters. Squeeze net model reduces the input module to smaller networks^19^. This small network helps in reducing the size and increasing compatibility. Squeeze net requires comparatively lesser processing time, reducing CPU inferences.

#### Resnet-18

Resnet stands for residual network. This network architecture is formed on the basis of VGG-16 network. But the layers in resnet-18 are short circuited. Figure 9 shows the circuit connection between the CNN layers. The concept followed here is to skip in between layers through short circuit connections. Hence this is some time called skip connections^20^. Which are responsible for further making residual blocks. Importance is given in mapping the residual block rather than the underlying layers themselves.

### Transfer learning

Transfer learning is an approach that consists of domains and tasks. The domain comprises of two components. A feature space ***H*** is defined in the domain D with marginal probability distribution P(H), where H = {h_1…,_ h_n},_ h_i_ ∈ ***H.*** A task in given specific domain D, specified as D = {***H***, P(F)}, consists of two components: a labelled space G and an objective predictive function ***f***:*** H→***G. Now for a new instance h, the prediction of corresponding label*** f***(k) is thru function ***f***. That is task T = {G,*** f***(k)}, is discovered from the training data consisting of pairs {h_i,_ g_i_}, where h_i_ ∈ ***H*** and g_i_ ∈ G

1 $${\varvec{T}}=\{{\text{G}},\mathbf{P}(\mathbf{G}/\mathbf{H})\}=\{\mathbf{G},\boldsymbol{ }{\varvec{f}}\left(\mathbf{k}\right)\}$$

When there is a known source domain D_S_ and task to learn **T**_**S**_, a target D_T_ and learning task T_L_ transfer learning accelerate the acquisition of anticipated target function ***f***_**T**_**(.)** in D_T_ using the knowledge in D_S_ and **T**_**S**_. The loss function generally represents the misclassification in predicted output to the input provided^8^. In transfer learning for classification-based optimization, the loss function is estimated as cross entropy. In machine learning cross entropy, is an approach used while the algorithms are developed to predict from the model. This is defined as given equation:

2 $$\mathbf{H}(\mathrm{ p},{\text{q}})=\sum_{x\epsilon X}p\left(x\right) Log q\left(x\right)$$

**Figure 7:**
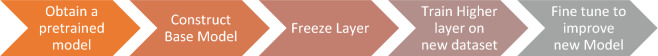
Transfer learning block diagram.

Here in the given equation x signifies the total number of values and p(x) categorizes the probability distribution in the real world and q(x) characterizes the probability of projected value in predicted environment. Overall, defining the loss function for predicted class^21^. Transfer learning implementation details is shown in Fig. 7, in the form of block diagram with the flow of action.

### Ethical and informed consent for the data used

The data used in the experiment were all obtained by the first author through field scouting.

## Experimental setup

**Figure 8:**
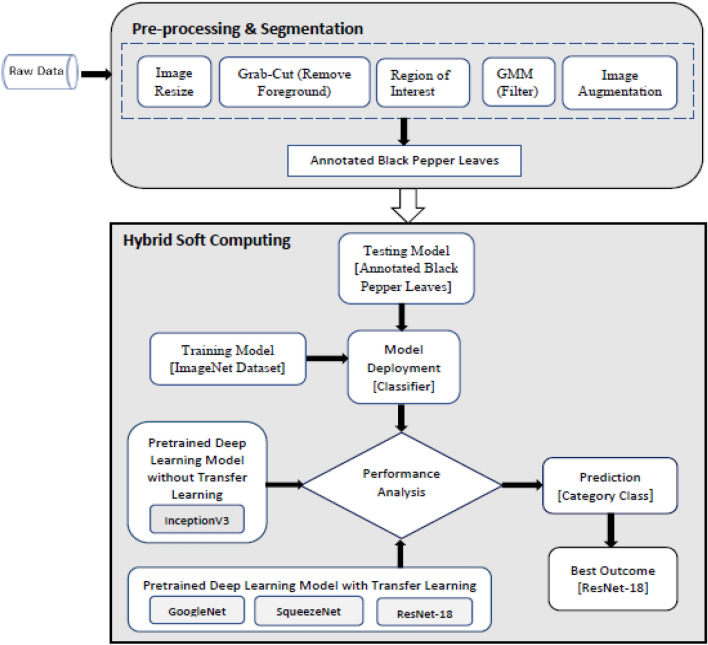
Illustration of the overall flowchart.

In this study, we introduce an ensembled state-of-the art deep learning method for predicting black pepper leaf diseases. The process is carried out in two phase, first phase is data preparation and second phase involve implementation of deep neural networks for diseases classification. Images are collected from the field and annotated. The annotated/labelled images are used for the experiment^22^. The dataset comprises of 1500 black pepper leaf images. Out of these, the best set of images are chosen for each disease category, i.e., for anthracnose, early stage phytophthora, phytophthora, slow wilt, yellowing and healthy leaf images. Hence, a total 600 images are labelled and saved^23^. The leaf images are chosen such that the diseases are clearly evident with high visibility on the leaf surface^24^. The insinuation of diseases in black pepper is that they are specific to the given region, and symptoms vary from one region to another. Hence, care is taken to correctly identify and diagnose the leaf diseases. Figure 8 illustrate flowchart of the overall model.

Image segmentation is done using grab-cut method. Grab cut is a very user-friendly and powerful algorithm for extracting the foreground objects from a marked region of interest. Image augmentation is necessary for the deep learning models to run effectively during the training phase^25^. Image augmentation relates to image data scaling and rotation. More information in the form of data variation enhances the performance of deep neural networks. Gaussian Mixture Model is used for further filtering background object.

**Figure 9:**
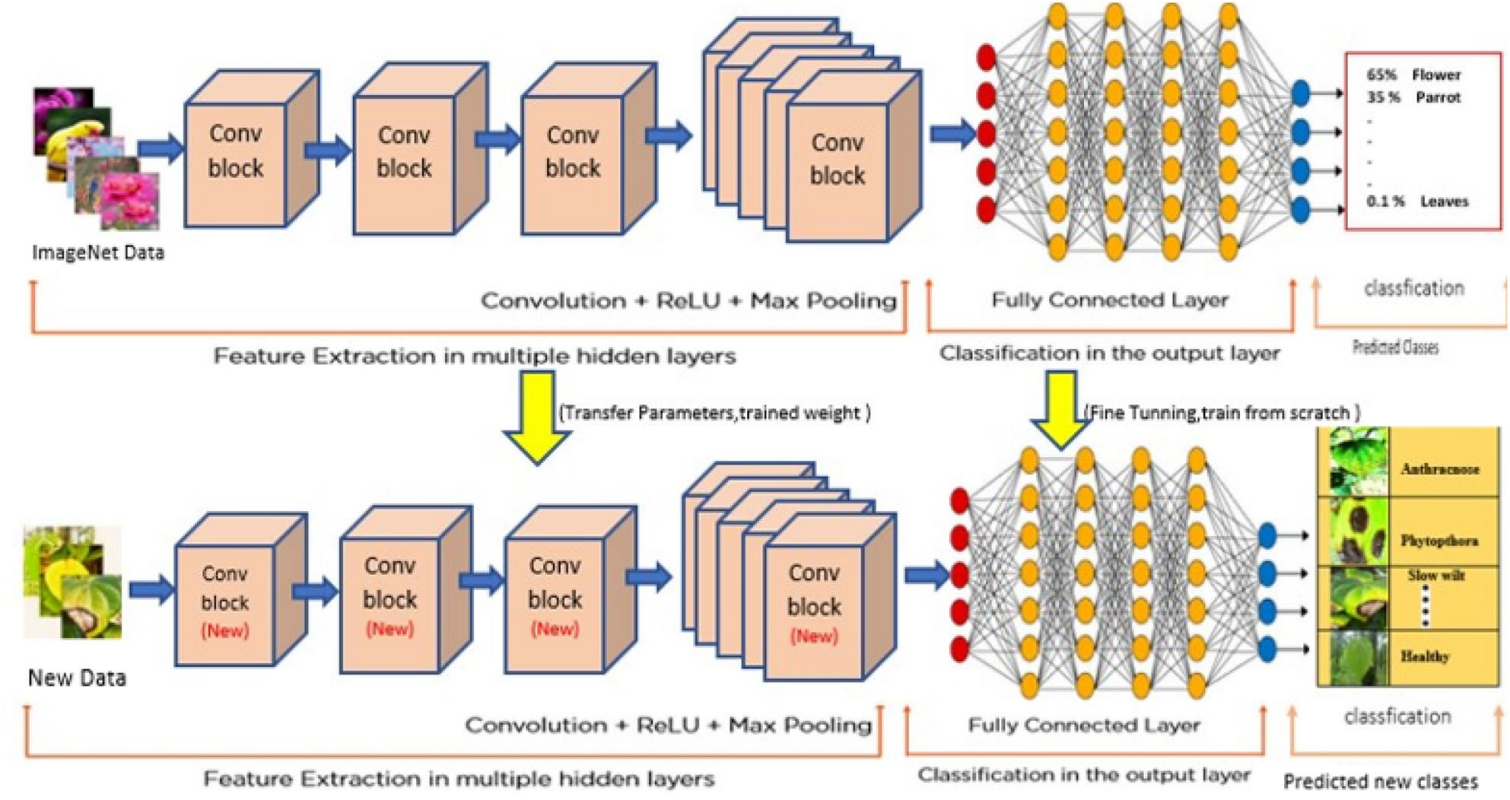
CNN implementation for the proposed Model.

**Figure 10:**
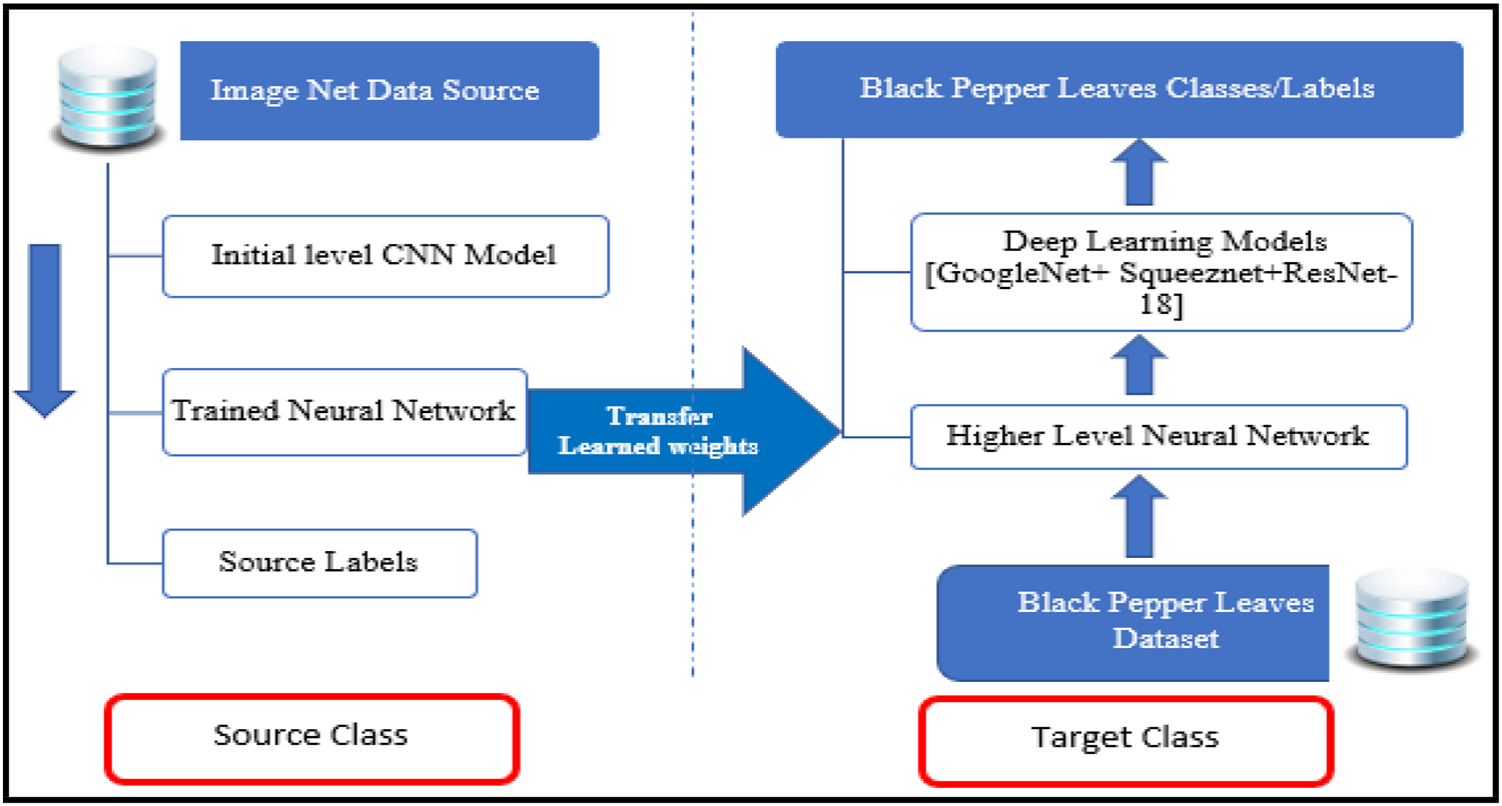
Proposed conceptual model.

Next, there was a need to import big data set to train deep neural networks so that these trained networks could be used for comparing and processing our novice data set. Hence, for training the CNN model, we have casted-off the most widely used image dataset, ImageNet. This repository has 10 billion unique images in its library^26,27^. This is beneficial for training and boosting the learning rate of deep neural network at the initial layer network. This data is loaded at the initial neural network layer in order to pretrain the neurons. To prevent misinterpretation, we gathered and considered the images from a single input source (one device) during the processes of segmentation and augmentation^28^. Images are resized to 256 × 256, the size expected by the input layer of each deep learning model. Large data sets are necessary for the deep learning performances; hence data augmentation is accoutered^29,30^. These are considered the new databases for transfer learning implementation. Figure 9 represents CNN implementation and 10 illustrates the flow of proposed conceptual methodology using transfer learning. Figure 10 embodies the conceptual model and the connections between implemented convolutional neural networks.

### Architecture for proposed deep learning implementation

Contemporary state-of-art deep neural network are the best models to utilize for any given task. Based on its accuracy, speed or any other parameters of importance, a DNN (deep neural network) might be classified as SOTA (State-of-the-art). The majority of computer vision fields, though, involve a trade-off between these measurements. In other words, even if a DNN is extremely quick, its accuracy may not be up to mark. Sometimes we can create a model with acceptable performance metrics, but it doesn’t have the necessary latency or throughput for a variety of applications, such as picture categorization and object detection^31^. In CNN, features are extracted from training data using the convolutional layer.

In this experiment, after meticulously training and working on new benchmark data set, four different deep neural networks: Inception V3, GoogleNet, SqueezeNet, and ResNet-18 are trained as state-of-the-art model for the black pepper leaf disease classification, using MATLAB R2023a software. First, the data is trained using Inception V3 individually with and without transfer learning, and the results are noted. Next an ensemble of state-of-the-art deep learning models that is GoogleNet, SqueezeNet and ResNet-18 are used with the optimizers; “adam”, “sgdm” and “rmsprop”. The hyperparameters used are data partitioning, loss functioning-batch size, initial learning rate schedule, learning rate, learn rate factor, number of epochs, validation frequency and bias learn rate factor.

The activation function used is ReLU, which is the most prevalent function in a deep neural network. Our classification problem is based on multiclass classification, hence SoftMax is employed. The code is written in python, importing all the necessary python packages in MATLAB. Further these networks are fine-tuned and learned higher-level layers are replaced by new layer including max-pooling, for the benchmark dataset classification. We have used cross entropy for improved classification probability and threshold cut to avoid overfitting. The accuracy obtained through transfer learning technique is very high. The proposed data is classified into: healthy and a set of diseased images. The six-leaf disease classification include Anthracnose, Early stage phytophthora, Slow wilt, Yellowing, Phytophthora and. Performance of the ensembled state-of-the-art network is measured by confusion metrics.

**Figure 11:**
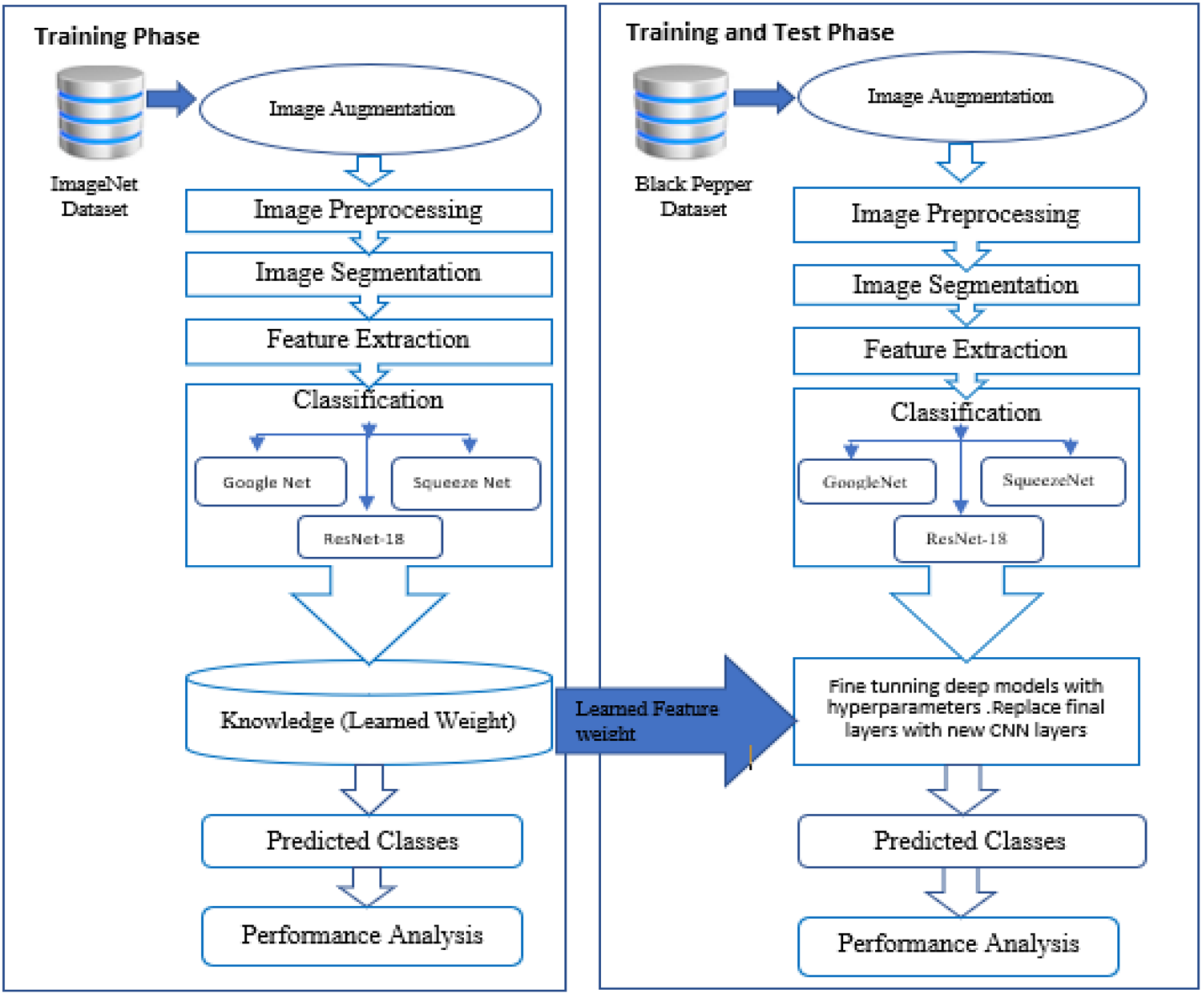
Architecture of proposed model.

Not much work is done in automating early leaf disease detection in black pepper leaves. Our approach is one of a kind for black pepper leaf disease detection. The implementation of deep learning algorithm has increased the performance of the classification model both in terms of accuracy and processing time using transfer learning technique. This work represents early disease classification and prediction with state-of-the-art deep learning models. This work represents implementation of high-end algorithms towards the automation of agricultural unit for the benefit of farmers and people working in fields. This is also an attempt to contribute to society, as the results obtained can be used in precision agriculture in the future. The architecture of the proposed work is depicted in Fig. 11.

**Figure:**
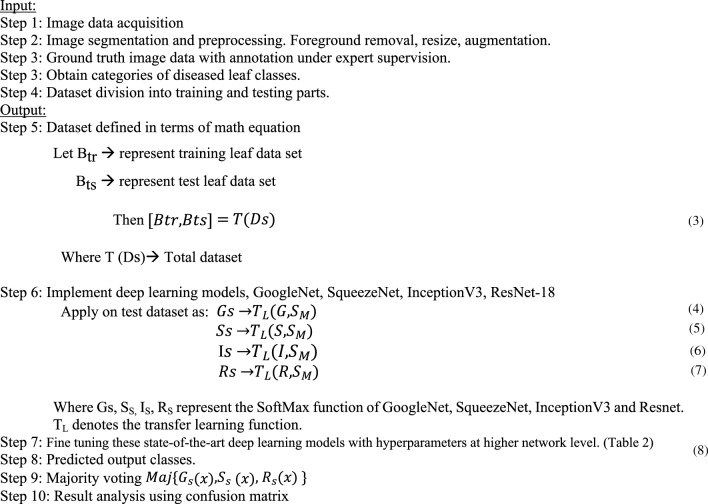
**Algorithm**: Implementing ensemble state-of-art deep learning model for prediction of black pepper leaves diseases using ConvNet.

#### Performance metrix

The efficiency of the proposed black pepper leaf disease detection is measured by the confusion matrix. Which gives performance measures based on true positives (TP), true negatives (TN), false positives (FP), and false negatives (FN). The confusion matrix is represented as in Table 1.

**Table 1:** Confusion matrix.

Confusion matrix	Predicted class	
Actual class	Positive	Negative
Positive	True Positive (TP)	False Positive (FP)
Negative	False Negative (FN)	True Negative (TN)

The performance that are computed through confusion matrix are specificity, sensitivity, precision, accuracy and F1-score.

#### Specificity

It is the performance measure, which represents the ratio of true negative to that of false positive and true negative.

9 $$Specificity=\left\{TN\right\}/\{FN+TN\}$$

#### Sensitivity/recall

It is the performance measure, which represents the ratio of true positive to that of true positive and false negative. It is also equivalent to recall.

10 $$Sensitivity(Recall)=\left\{TP\right\}/\{TP+FN\}$$

#### Precision

Precision represents the ratio of true positive to that of true positive and false positive. It is also equivalent to recall.

11 $$Precision=\left\{TP\right\}/\{TP+FP\}$$

#### Accuracy

This performance measure is defined as the ratio of sum of true positive and true negative to that of total sum of true positive, false positive, true negative and false negative.

12 $$Accuracy=\left\{TP+TN\right\}/\{TP+TN+FP+FN\}$$

*F1-score*: This performance metric is the harmonic mean of recall and precision. It is measure of the accuracy. Represented as given equation.

13 $${F}_{1}=\left\{2*Precision *Recall\right\}/\left\{Precision +Recall\right\} =(2*TP\}\{2*TP +FP+FN \}$$

The *hyperparameter* values tuned for designing neural network are coded as follows:

In order to fine tune our model, the hyperparameters are manually coded in python with given parameters.

For training and testing two sets were considered with 70:30 and 60:40 ratio. But 70:30 gave better result and 60:40 division was not a better choice. So, the result considered were for 70/30 sets i.e. 70% training and 30% testing data.

- Data partition = 1. Training = 70% and Testing 30% and 2. Training = 60% and Testing 40%
- Input image size = 1.224 × 224 × 3, for Inception V3 Model and 2.227 × 227 × 3, for Google Net, ResNet-18 and Squeeze Net models.
- Mini-batch size = 200
- initial learning rate = 0.001 and 0.0001
- learning rate schedule = Time decay
- number of epochs = 30
- Loss function = 10
- bias learn rate factor = 10
- validation patience = 40
- validation frequency = 10
- performance metrics = Confusion Metrix
- Optimization Algorithms = Adam, sgdm, rmsprop

## Result and discussions

**Figure 12:**
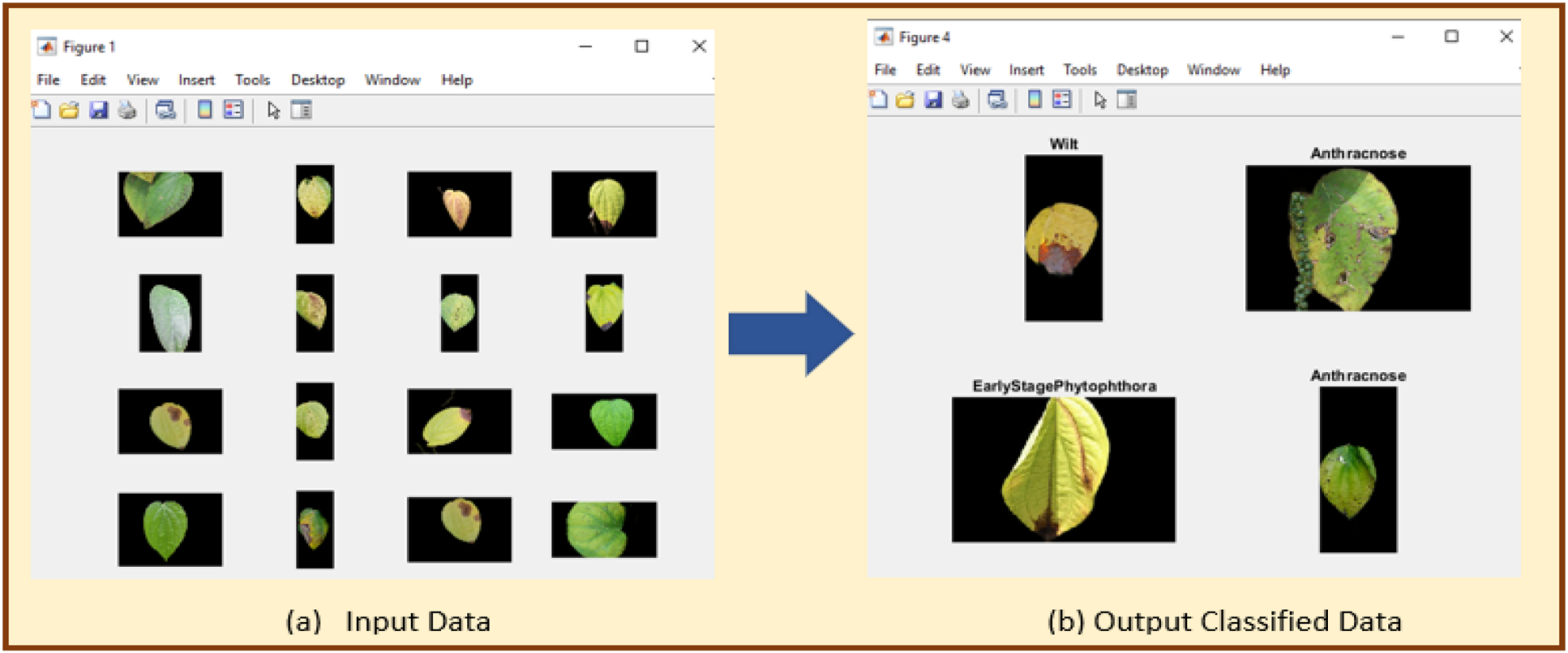
Predicted black pepper leaves diseases (**a**) Input, (**b**) Output.

We have used MATLAB R2023a for our deep neural network implementation. The software used to design neural networks is Python. For image preprocessing GIMP software is used. The deep neural network is customized accordingly to get better performance. This is done by manually adding layer-by-layer networks by dropping and adding network layers in the MATLAB R2023a deep neural network designer tool. The hyper-parameters, including normal network parameters, are added with trial and error to find the most suitable one. The main matrix values in each designer network are maintained as compared with the standard values. An ensemble of deep learning models is used for six black pepper leaf disease varieties. These leaf diseases are saved in different directories as a set of training and testing dataset modules. Figure 12 depicts MATLAB implementation of predicted Black Pepper Leaves Diseases (a) Input, (b) Output. Python code is written by downloading all the necessary packages required for the proposed model and functions are added to execute four deep learning models. These functions are in-built and available with the tool. Since we are using transfer learning, the proposed algorithm was first trained (executed) on ImageNet (online) data and then the last layers of these convolutional neural networks were replaced by a new network by simply deleting the upper layer and dropping a new upper layer. Then the algorithm was re-executed for the new black pepper leaf dataset. Table 2 shows the various hypermeters used in the experiment and their definitions and values. This is also explained in the performance matrix headline in this paper.

**Table 2:** Hyperparameters used in the experiment.

S. no.	Hyperparameters	Description	Value/parameters
1	Data partition	It relates to the division made w.r.t training and testing dataset	Data divided as ratio of: 1. Training = 70% and Testing 30% 2. Training = 60% and Testing 40%
2	Input image size	The image size required by the model being trained	1. 224 × 224 × 3 2. 227 × 227 × 3
3	Loss function	Relates to error occurred with given value to that of algorithm output	0 to 1.00
4	Mini-batch size	Normally the batch sizes considered are power of 2. Not recommended for dataset less < 2000	64, 128, 256, 512 etc.
5	Initial learning rate	It is the value when neural network starts learning	0.001/0.0001
6	Learning rate schedule	A parameter to adjust networks learning to that of predefined value	1. Time decay 2. Step decay 3. Exponential decay
7	Number of epochs	Relates to the iteratively working/training on entire batch of data	10, 25, 30
8	Learn rate factor	It is non-negative scalar value. Where learning rate of specific parameter is compared to global learning rate	10
9	Bias learn rate factor	Rate at which neural network learns	10
10	Validation patience	It is the maximum tries the algorithm makes w.r.t epoch for performance improvement	40
11	Validation frequency	It is the lowest value by which the given model is to validated	10
12	Performance metrics	Measure of the classification performance of machine learning algorithms	Confusion Metrix
13	Optimizers	A function that familiarizes the neural network’s weights and learning rate	Adam, Sgdm, Rmsprop

**Figure 13:**
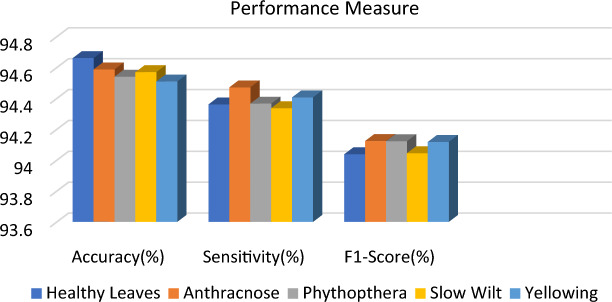
Performance measure of Inception V3.

**Figure 14:**
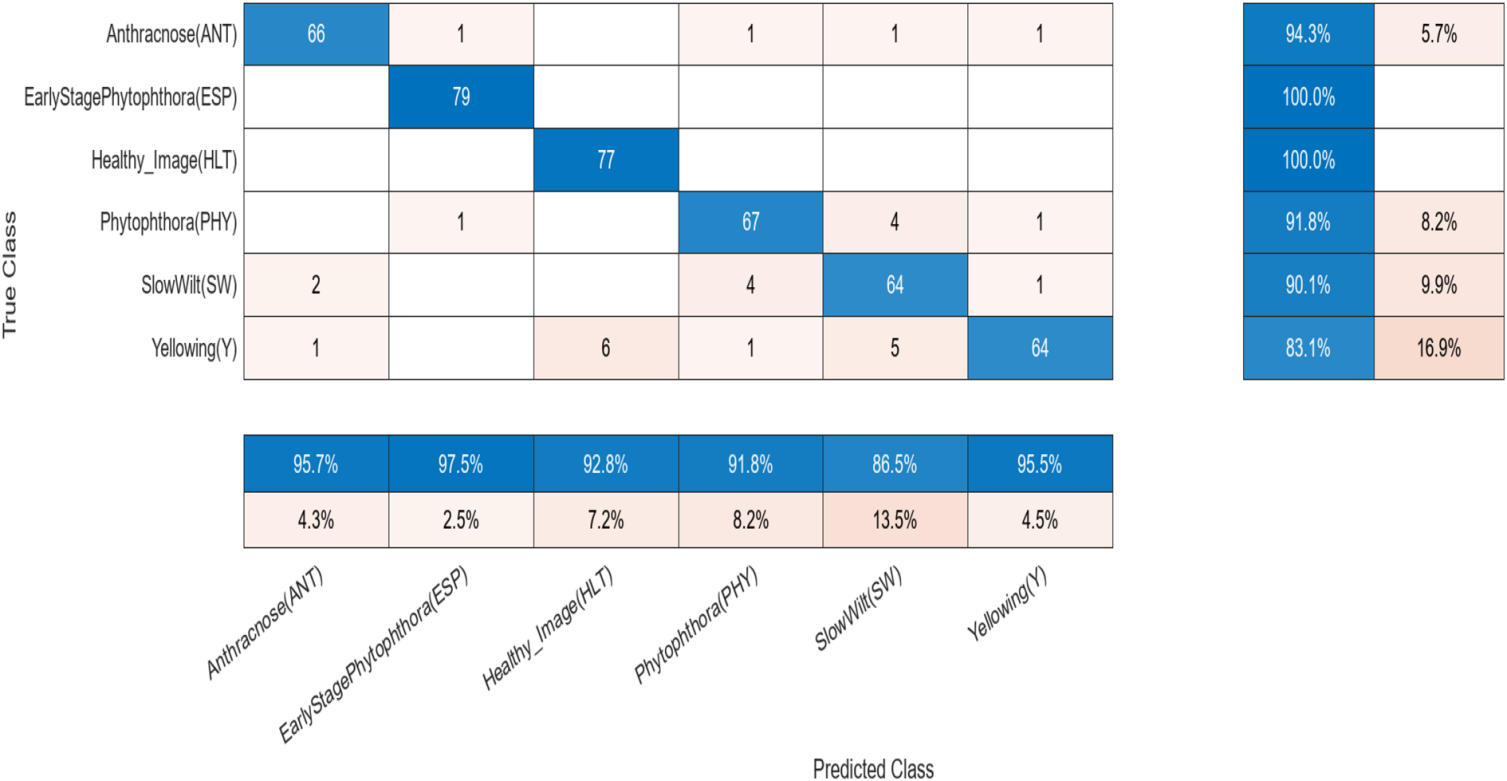
Confusion matrix (black pepper leaf disease, state-or-the-art implementation).

**Figure 15:**
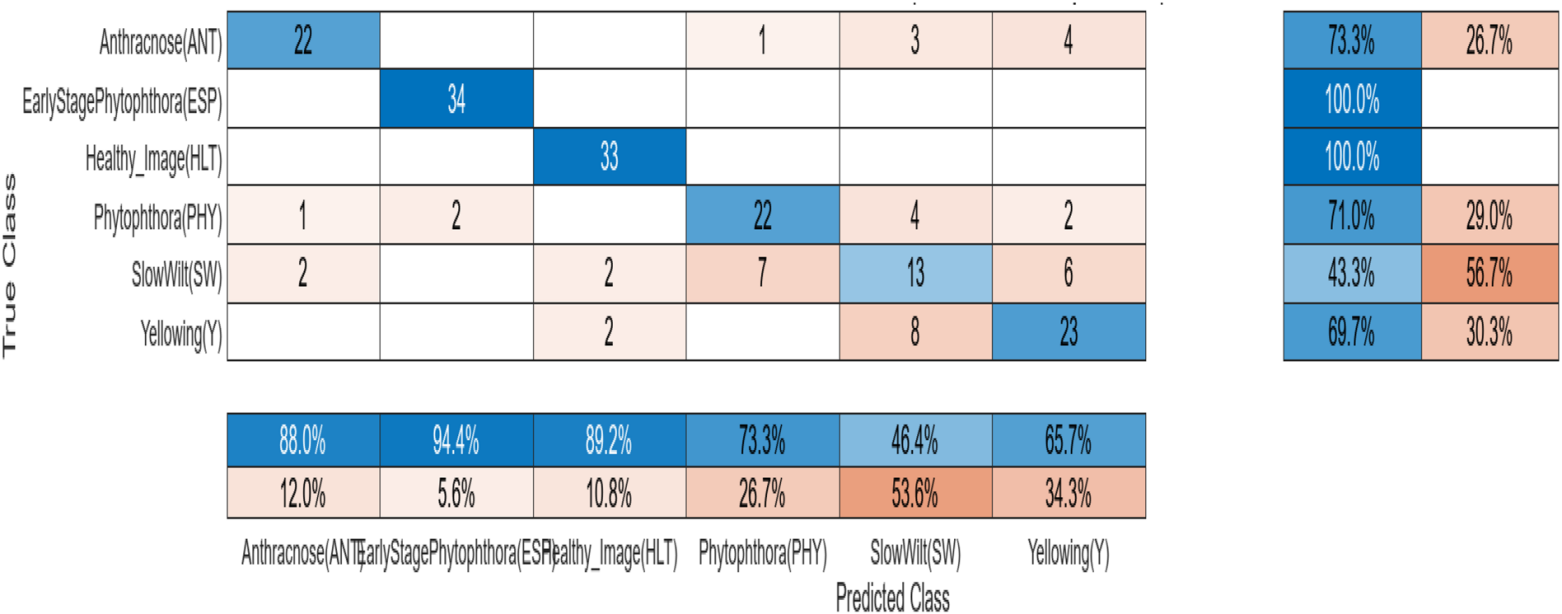
Confusion matrix (black pepper leaves).

**Figure 16:**
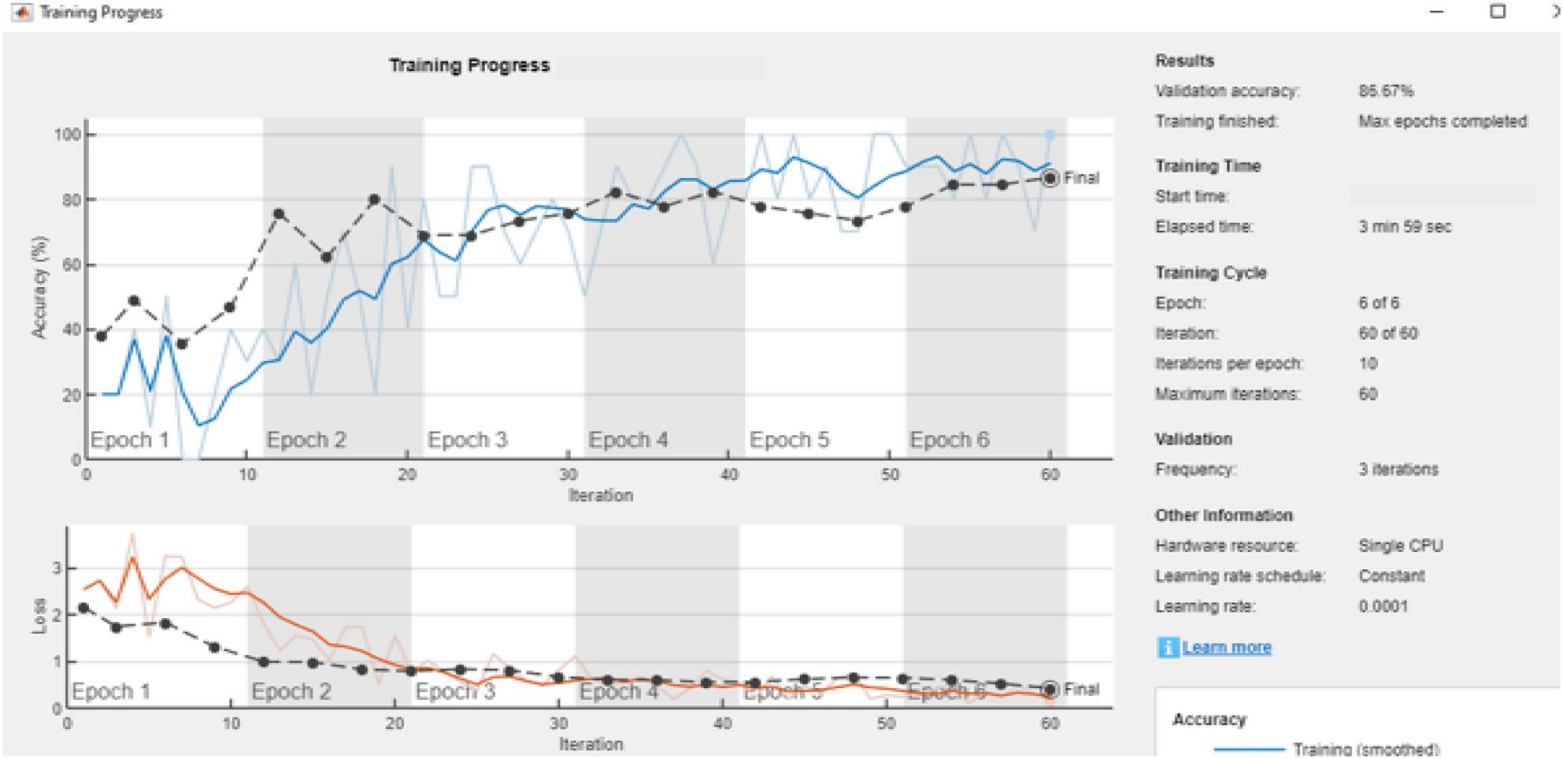
Accuracy and loss graph Inception V3 implementation.

**Figure 17:**
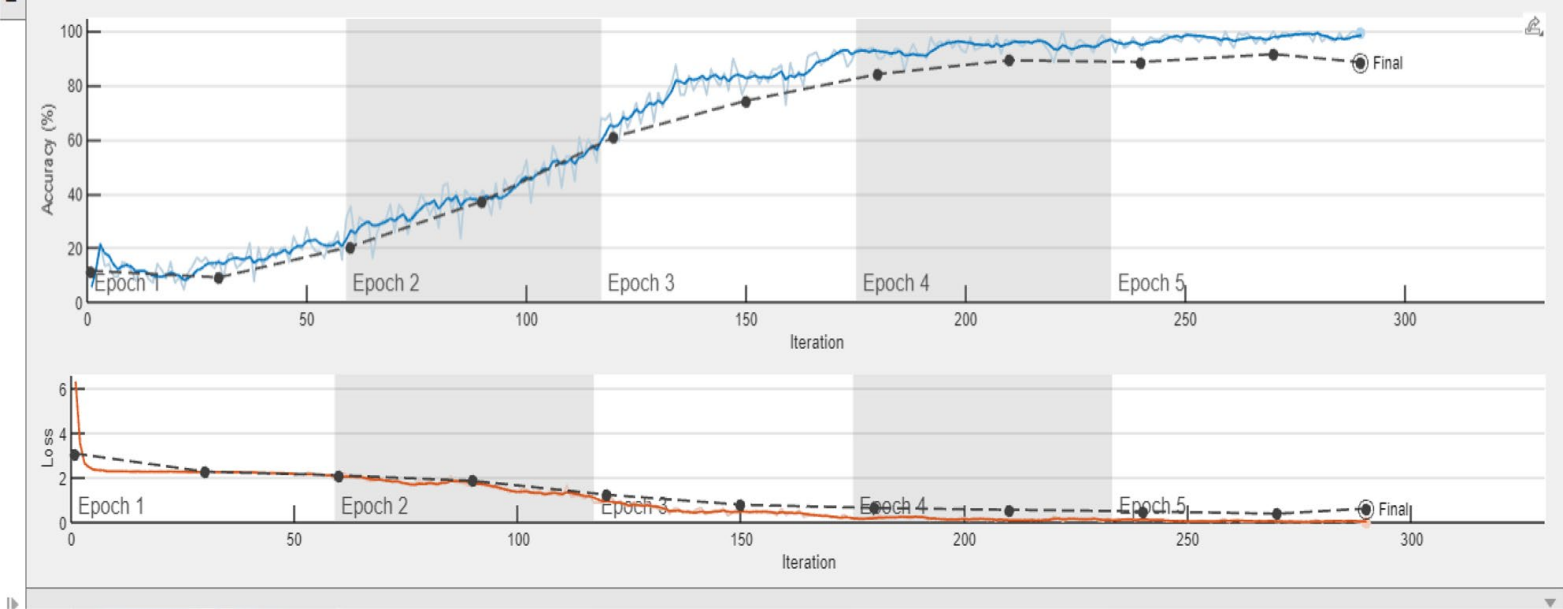
Accuracy and loss graph for the black pepper leaves disease prediction.

**Figure 18:**
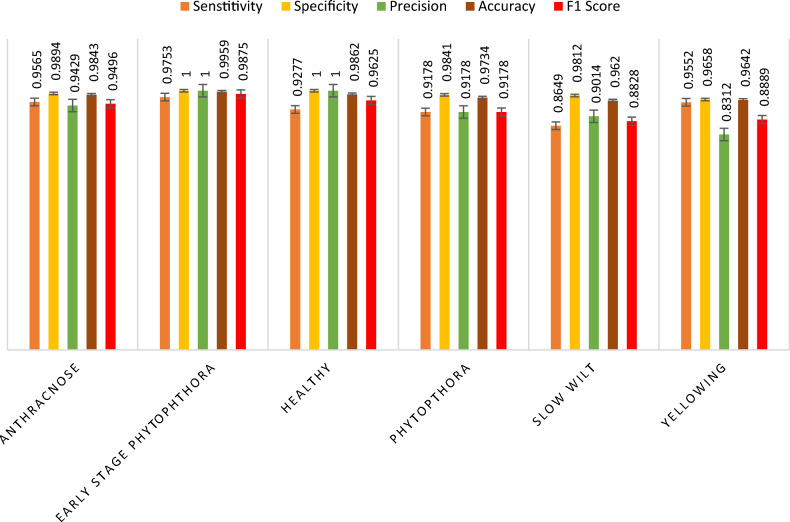
Performance measure of state-of-the-art deep neural network.

**Figure 19:**
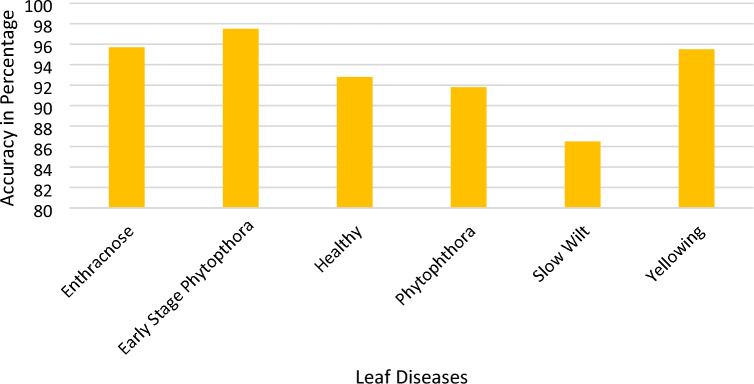
Accuracy in percentage (%) of predicted black pepper leaf diseases.

**Figure 20:**
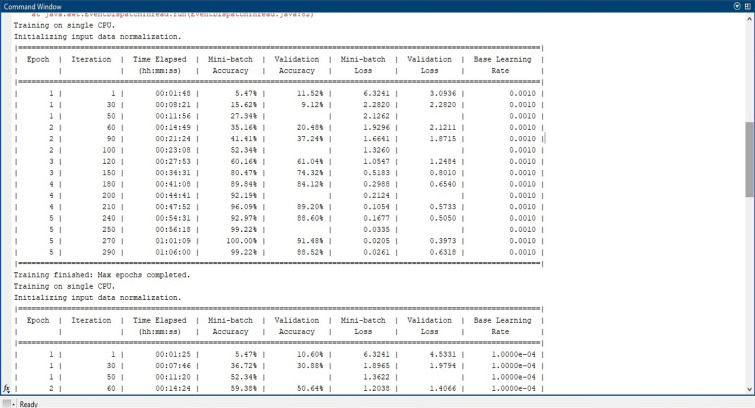
Output sample with execution time.

A comparative analysis is done with the other models given in the various research papers shown in Table 3. A comparison is also shown in Table 4 with the proposed model's accuracy in the given experiment. The stage-wise steps are specified and explained through the algorithm: Implementing ConvNets, state-of-art deep learning model for prediction of black pepper leaves diseases. A total of nine different model combinations are made with three different optimizers: “Adam”, “sgdm,” and “rmsprop." The images are resized at the pre-processing stage as required by the deep learning model algorithm, i.e., 224 × 224 × 3 and 227 × 227 × 3, etc. The learning rate used in the algorithm is between 0.001 and 0.0001. Image augmentation and segmentation are done during the preprocessing stage. This helped in having better accuracy and an F1 score of 0.78 to 0.98. Ensemble deep learning approach with transfer learning technique resulted in high prediction of black pepper disease classification from 65 to 99%. This is seen with the Inception V3 implementation in both cases. Figure 13 displays Inception-V3 performance. Tables 5 and 6 exhibit architecture layer details and analysis results of Inception V3. The performance of the state-of-the-art deep learning model is measured through a confusion matrix. The resulting confusion matrix in the MATLAB tool is shown in Figs. 13 and 14. Where the comparison is shown for training as well as testing data. Figures 15 and 16 demonstrate the accuracy graphs for the implemented models. The performance measure acquired by the three models “GoogleNet”, “SqueezeNet,” and “ResNet-18” is shown in Figs. 17 and 18. The values are computed through a confusion matrix. The statistic is displayed in Table 7. It is also observed that the performance of the deep learning models varies with the optimizers used. GoogleNet works well with rmsprop and sgdm. SqueezeNet works better with the sgdm optimizer. ResNet-18 results in high accuracy with the rmsprop optimizer. The proposed modified Res-18 outperforms all other deep models used in the experiment. Accuracy in percentage of predicted black pepper leaf diseases is shown in Fig. 19. The output sample with execution time is shown in Fig. 20. But the outcome may vary for different datasets other than ImageNet and the proposed diseased black pepper leaf dataset. The efficiency of each available high-end deep neural network algorithm depends on experimentation and analysis.

**Table 3:** Comparison result of other deep learning models.

Reference	Classification model	Type of Leaf	Accuracy
Ganatra, N. and Patel, A^7^	Random Forest	Multiple	73.38%
Saleem MH^8^	Xception architecture	Multiple	91.86
Shreya Ghosal^9^	VGG-16	Rice Leaf	92.46%
Hassan, S k Mahmudul^11^	InceptionResNetV2	Plant Village Dataset	97.02%
Arvind Krishnaswamy R^13^	VGG-16	Selected Leaf	90
Santosh Kumar Up^16^	CNN	Paddy leaf	97.2%
Saleem, Muhammad H^20^	Deep Neural Network	Tomato Leaf	95.76%
Geetharamani, G^23^	Deep CNN	Plant Leaf Dataset	96.46%
Chen J, Zeb A^32^	Es-MbNet	Plant Village dataset	99.37%
Nanehkaran YA^33^	CNN	Online and offline	91.33 ± 3.34

**Table 4:** Comparison of state of art deep learning approach for black pepper leaves.

Deep neural network	Classification model	Leaf type	Accuracy (%)
Inception V3	Inception V3 (without transfer learning)	Black Pepper Leaf	62.3
Inception V3	Inception V3 (with transfer learning)	Black Pepper Leaf	94.6
GoogleNet	GoogleNet (with transfer learning)	Black Pepper Leaf	99.5
SqueezeNet	SqueezeNet (with transfer learning)	Black Pepper Leaf	99.4
Proposed Model	ResNet-18 (with transfer learning)	Black Pepper Leaf	99.67

**Table 5:** Inception V3 detailed parameter and architecture layer.

Type	Patch size/stride	Output size	depth	#1 × 1	#3 × 3 reduce	#3 × 3	# 5 × 5 reduce	#5 × 5	Pool proj	params	ops
convolution	7 × 7/2	112 × 112 × 64	1							2.7 k	34 M
max pool	3 × 3/2	56 × 56 × 64	0								
convolution	3 × 3/1	56 × 56 × 192	2		64	192				112 K	360 M
max pool	3 × 3/2	28 × 28 × 192	0								
inception(3a)		28 × 28 × 256	2	64	96	128	16	32	32	159 K	128 M
Inception(3b)		28 × 28 × 480	2	128	128	192	32	96	64	380 K	304 M
max pool	3 × 3/2	14 × 14 × 480	0								
inception(4a)		14 × 14 × 512	2	192	96	208	16	48	64	364 K	73 M
inception(4b)		14 × 14 × 512	2	160	112	224	24	64	64	437 K	88 M
inception(4c)		14 × 14 × 512	2	128	128	256	24	64	64	463 K	100 M
inception(4d)		14 × 14 × 528	2	112	144	288	32	64	64	580 K	119 M
inception(4e)		14 × 14 × 832	2	256	160	320	32	128	128	840 K	170 M
max pool	3 × 3/2	7 × 7 × 832	0								
inception(5a)		7 × 7 × 832	2	256	160	320	32	128	128	1072 K	54 M
inception(5b)		7 × 7 × 1024	2	384	192	384	48	128	128	1388 K	71 M
avg pool	7 × 7/1	1 × 1 × 1024	0								
dropout(40%)		1 × 1 × 1024	0								
linear		1 × 1 × 1000	1							1000 K	1 M
softmax		1 × 1 × 1000	0

**Table 6:** Inception V3 analysis result.

Name	Activations	Type	Learnable
Inception_5b_1 × 1 384 1 × 1 × 832 convolutions with stride [1 1] and padding [0 0 0 0]	7 × 7 × 384	Convolution	Weights 1 × 1 × 832 × 384 Bias 1 × 1 × 384
Inception_5b-relu_1 × 1 ReLU	7 × 7 × 384	ReLU	-
Inception_5b-5 × 5_reduce 48 1 × 1 × 832 convolutions with stride [1 1] and padding [0 0 0 0]	7 × 7 × 48	convolution	Weights 1 × 1 × 832 × 48 Bias 1 × 1 × 48
Inception_5b-relu_5 × 5_reduce ReLU	7 × 7 × 48	ReLU	–
Inception_5b-relu_5 × 5_reduce 128 5 × 5 × 48 convolutions with stride [1 1] and padding [2 2 2 2]	7 × 7 × 128	Convolution	Weights 5 × 5 × 48 × 128 Bias 1 × 1 × 128
Inception_5b-relu_5 × 5 ReLU	7 × 7 × 128	ReLU	–
Inception_5b-relu_3 × 3_reduce ReLU	7 × 7 × 192	ReLU	–
Inception_5b-3 × 3 384 3 × 3 × 192 convolutions with stride [1 1] and padding [1 1 1 1]	7 × 7 × 384	Convolution	Weights 3 × 3 × 192 × 384 Bias 1 × 1 × 384

**Table 7:** Statistic measure for state -of -the-art deep learning model.

Measure	Sensitivity/recall	Specificity	Precision	Accuracy	F1 Score
Derivations/predicted classification	TPR = TP/(TP + FN)	SPC = TN/(FP + TN)	PPV = TP/(TP + FP)	ACC = (TP + TN)/(P + N)	F1 = 2TP/(2TP + FP + FN)
Anthracnose (ANT)	0.9565	0.9894	0.9429	0.9843	0.9496
EarlyStagePhytophthora (ESP)	0.9753	1.0000	1.0000	0.9959	0.9875
Healthy (HLT)	0.9277	1.0000	1.0000	0.9862	0.9625
Phytophthora (PHY)	0.9178	0.9841	0.9178	0.9734	0.9178
Slow Wilt (SW)	0.8649	0.9812	0.9014	0.9620	0.8828
Yellowing(Y)	0.9552	0.9658	0.8312	0.9642	0.8889

This research focuses on the early diagnosis of black pepper plant leaf diseases with the comprehension of computer vision. This is the first attempt of its kind to detect black pepper leaf disease using a deep learning approach. The proposed technique helps in predicting early symptoms of various black pepper leaf diseases. The state-of-the-art deep neural networks are implemented successfully on the newly created leaf dataset with an accuracy of 99.7%. For this study, five types of black pepper leaf diseases were considered: anthracnose, early stage phytophthora, quick wilt, yellowing, and slow wilt, as well as healthy leaves. The accuracy alters with respect to optimizers used. Deep learning models work well when data is available in millions. ResNet-18 provided high accuracy 99.7% when compared with other models.

This technique can be further developed as an app for smart phones. This work proposes a solution towards agricultural intelligence for predicting diseases remotely with the application of high potential deep neural network in precision agriculture. An automated leaves prediction system can also be used by non-botanical experts to quickly identify early plants diseases quite effortlessly. Deep learning can aid the task of remotely sensing fields where human intervention is vulnerable.

In the majority of circumstances, the traditional approach to diagnosis disease is similar. This may lead to misinterpretation about the diseases if it is unknown. Which may further tip to uneven application of pesticides in the fields, resulting in crop loss. Though diseases can be predicted based on climate conditions, their symptoms cannot be judged. With the help of the proposed method early disease predictions is possible. This will aid in reduction of unwanted pesticides distribution. A web-based or mobile-based computer system for the automatic classification of medicinal plants such as black pepper will help the local population to improve their knowledge of medicinal plants.

## Future work

In the future, region-growing segmentation techniques can be used interactively for profound disease spot detection in leaves. Bountiful advanced deep learning technologies could be explored, such as VGG-101, VGG-S, U-Net, V-Net, Yolo, and Fuzzy Logic, for leaf disease detection, classification and prediction. Zone-based or global feature-based feature extraction techniques could also be explored. Synthetically developed image data can also be used in future, provided the required input data is in few hundreds.

## Data Availability

The data used to support the given experiment are available on request through the first author’s Email.
