# Author Correction: Early stage black pepper leaf disease prediction based on transfer learning using ConvNets

**DOI:** 10.1038/s41598-024-55982-x

**Published:** 2024-03-04

**Authors:** Anita S. Kini, K. V. Prema, Smitha N. Pai

**Affiliations:** 1 Department of Computer Science and Engineering, Manipal Institute of Technology, Manipal Academy of Higher Education (MAHE), Manipal, India; 2 Department of Computer Science and Engineering, Manipal Institute of Technology Bengaluru, Manipal Academy of Higher Education (MAHE), Manipal, India; 3 Department of Information and Communication Technology, Manipal Institute of Technology, Manipal Academy of Higher Education (MAHE), Manipal, India

Correction to: *Scientific Reports* 10.1038/s41598-024-51884-0, published online 16 January 2024

The original version of this Article contained an error in Affiliation 2. Affiliation 2 was incorrectly given as ‘Department of Computer Science and Engineering, Manipal Institute of Technology, Manipal Academy of Higher Education, Bangalore, India’.

The correct affiliation is listed below:

Department of Computer Science and Engineering, Manipal Institute of Technology Bengaluru, Manipal Academy of Higher Education (MAHE), Manipal, India.

The original Article has been corrected.

